# Real World Application of Stenting of Unprotected Left Main Coronary Stenosis: A Single-Center Experience

**DOI:** 10.4021/cr165w

**Published:** 2012-05-20

**Authors:** Calvin C. Leung, Timothy C. Ball, Mandeep S. Sidhu, James T. DeVries, John E. Jayne, John F. Robb, Aaron V. Kaplan, Jeremiah R. Brown, David J. Malenka, Craig A. Thompson

**Affiliations:** a Section of Cardiology, Department of Medicine, University of South Florida, Tampa, FL, USA; b Section of Cardiology, Department of Medicine, Dartmouth-Hitchcock Medical Center, Lebanon, NH, USA; c The Dartmouth Institute for Health Policy and Clinical Practice, Lebanon, NH, USA; d Yale University School of Medicine, New Haven, CT, USA

**Keywords:** Cardiac catheterization, Left main coronary artery, Percutaneous intervention, Stenting

## Abstract

**Background:**

The aim of this study was to summarize our single-center real-world experience with percutaneous coronary intervention (PCI) stenting of unprotected left main coronary artery (ULMCA). PCI-stenting of the ULMCA, while controversial, is emerging as an alternative to coronary artery bypass graft (CABG) surgery in select patients and clinical situations.

**Methods:**

Between January 2005 and December 2008, PCI-stenting was performed on 125 patients with ULMCA lesions at our institution. Clinical and procedural data were recorded at the time of procedure, and patients were followed prospectively (mean 1.7 years; range 1 day-4.1 years) for outcomes, including death, myocardial infarction (MI), and target vessel revascularization (TVR).

**Results:**

The majority of cases were urgent or emergent (82.5%), 50.4% of patients were non-surgical candidates, and 63.2% had 3 vessel disease. Many emergent patients presented in shock (62.1%), were not surgical candidates (89.7%), and had high mortality (20.7% in-hospital, 44.8% long-term). Mortality in the elective group was 6.3%. Cumulative death and TVR rates were 28.8% and 13.6%, respectively. Independent predictors of mortality were ejection fraction (EF) ≤ 35% (HR 2.4, CI 1.1 - 5.4) and left main bifurcation (HR 2.7, CI 1.2 - 5.7).

**Conclusions:**

PCI-stenting is a viable option in patients with LMCA disease and extends options to patients who are poor candidates for CABG. Elective PCI in low-risk CABG patients results in good long-term survival. Cumulative TVR is 13.6%. EF ≤ 35% and left main bifurcation are independently associated with increased mortality.

## Introduction

Left main coronary artery (LMCA) stenoses are found in 3-10% of patients undergoing coronary angiography [1, 2]. Medical treatment results in one year mortality of 21% [3] and three year mortality of 30-40% [4-7]. Coronary artery bypass graft surgery (CABG) leads to improved long-term survival [4-7] and continues to be the recommendation for the treatment of unprotected LMCA lesions [8]. ULMCA PCI has been upgraded to a Class IIb recommendation according to American College of Cardiology/American Heart Association guidelines for patients not suitable for CABG.

## Methods

### Study design

This is a prospective registry study looking at patients in a “real world” application undergoing PCI-stenting of LMCA lesions at Dartmouth-Hitchcock Medical Center, an academic rural regional medical center. Patients were identified at the time of the procedure with demographic, clinical, angiographic, and procedural data and followed prospectively in the Dartmouth Dynamic Registry, which has been approved by the Institutional Review Board (Dartmouth Center for the Protection of Human Subjects).

### Study population

Patients were included in the study if they had PCI-stenting done on significant de novo unprotected LMCA lesions (> 50% diameter) between January 2005 and December 2008. An unprotected lesion was defined as one where the patient either had not had prior CABG, had a bypass but no patent grafts to the left coronary system, or the bypass graft was to the right coronary artery only. In this time frame, 221 interventions were done to LMCA lesions, 95 cases were excluded for being protected, and one case was excluded as it was a thrombectomy without any stenting. The final cohort was 125 patients. Only one patient had a prior CABG, which was to the right coronary artery.

The decision to perform PCI-stenting versus CABG was dependent upon patient co-morbidities, urgency, adequate surgical targets, patient preference, or physician preference. In the majority of cases, there were discussions between the cardiologists and cardiothoracic surgeons regarding optimal therapy and surgical candidacy. The use of intravascular ultrasound (IVUS), intra-aortic balloon pump (IABP), and treatment of lesions including bifurcation lesions were left up to the preference of the interventional cardiologists.

Patients were followed for clinical outcomes: new MI, target vessel revascularization (TVR), any revascularization, and death.

### Statistical analysis

Means for continuous variables are calculated for different groups (± 1 SD) and compared with Student’s t-tests or test of trend where appropriate. Binary variables are presented as percentages and compared with chi-square testing or Fisher’s exact test where appropriate. Statistical significance was established at the 0.05 alpha level. Surgical and PCI predicted risk scores were calculated using published models [21-23]. Kaplan-Meier outcomes and survival curves were done with adjustment by the Ghali Method with analysis by log-rank test. Cox’s proportional hazard model was used to determine crude and adjusted hazard ratios with Breslow Method for ties, adjusting for the variables with significant crude hazard ratios: EF ≤ 35%, left main bifurcation, IABP use, Parsonnet score, Euroscore, cardiogenic shock, positive troponin, and emergent priority. Analyses were performed using Stata 10.0 (College Station, TX).

## Results

### Study participants

**Figure 1:**
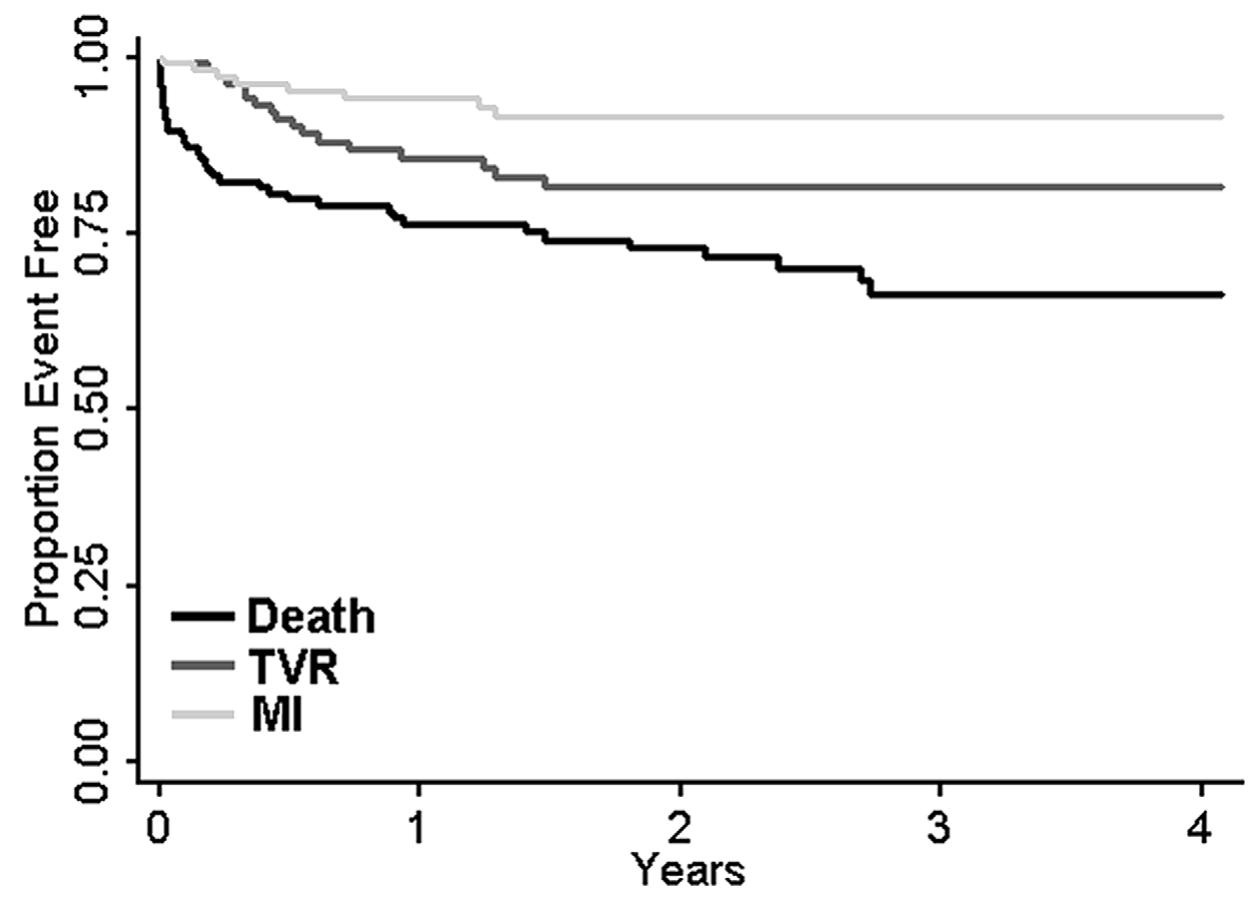
Kaplan-Meier graph of outcomes for left main percutaneous coronary intervention. Kaplan-Meier outcomes curves for individual and composite outcomes: death, target vessel revascularization (TVR), and myocardial infarction (MI) for patients undergoing left main coronary artery intervention.

Patient characteristics are presented in Table 1, and procedural data is presented in Table 2. Continuous variables are presented with means ± 1 SD. Mean patient follow-up was 1.8 years with longest follow-up of 4.1 years. A prior history of CAD was present in 58% of patients: one patient had a prior CABG and 21.6% of patients had a prior PCI, 50.4% of patients were non-surgical candidates, 3-vessel disease was present in 63.2% of individuals. Cases were either urgent or emergent 87.2% of the time. By using an additive Euroscore value of 6 or more, 74.4% of patients were considered to be at high surgical risk. On average, 2.69 lesions were treated per case with an average of 3.0 stents used. Survival was 89.6% at 30 days, 76.8% at 1 year, and 71.2% at 4 years (Fig. 1) with a cumulative TVR rate of 13.6%.

**Table 1:** Characteristics for All Patients and Comparison of Bifurcation and Non-Bifurcation Cohorts

Variables	All	Bifurcation	Non-bifurcation	P-value
Number (N = 125)	125 (100%)	64 (51.2%)	61 (48.8%)	
Demographics				
Age (years)	73.7 ± 10.9	73.9 ± 11.0	73.5 ± 10.9	0.85
Gender (female; %)	40.8	39.1	42.6	0.69
Body mass index	28.2 ± 6.7	28.3 ± 6.6	28.0 ± 6.9	0.79
Co-morbidities				
Chronic obstructive pulmonary disease (%)	20.0	18.8	21.3	0.72
Peripheral vascular disease (%)	34.4	37.5	31.2	0.46
Baseline creatinine (mg/dL)	1.4 ± 1.1	1.3 ± 0.8	1.5 ± 1.4	0.26
Creatinine >2 mg/dL (%)	10.4	7.8	13.1	0.33
Risk Factors				
Diabetes (%)	36.8	31.3	42.6	0.19
Smoking history (%)	52.0	48.4	55.7	0.41
Hypertension (%)	79.2	81.3	77.0	0.56
Hypercholesterolemia (%)	72.8	71.9	73.8	0.81
Family history of CAD (%)	36.0	39.1	32.8	0.47
Previous Cardiac History and Cardiac Function				
History of CAD (%)	58.4	62.5	54.1	0.34
Prior thrombolysis (%)	3.2	1.6	4.9	0.29
Prior PCI (%)	21.6	28.1	14.8	0.07
Prior CABG (%)	0.8	0.0	1.6	0.30
EF	49.3 ± 15.2	52.2 ± 13.8	46.1 ± 16.0	0.02
EF ≤ 35% (%)	23.2	15.6	31.1	0.04
Mitral regurgitation ≥ 2+ (%)	26.4	18.8	24.6	0.43
Indications for intervention				
Stable angina (%)	16.0	17.2	14.8	0.71
ST-elevation MI (%)	10.4	10.9	9.8	0.84
Non-ST-elevation MI (%)	60.0	60.9	59.0	0.83
Unstable angina (%)	8.8	7.8	9.8	0.69
Cardiogenic shock (%)	18.4	17.2	19.7	0.72
Non-surgical candidate (%)	50.4	53.1	47.5	0.53
Parsonnet score	22.1 ± 22.6	22.1 ± 21.9	22.2 ± 23.6	0.98
Euroscore (Logistic)	17.0 ± 16.6	15.7 ± 15.8	18.3 ± 17.5	0.39
Euroscore (Additive)	8.5 ± 3.9	8.3 ± 3.7	8.7 ± 4.2	0.54
Northern New England In-hospital Predicted Mortality Probabilities for PCI	13.3 ± 21.8	10.8 ± 18.3	16.0 ± 24.8	0.18
Northern New England In-hospital Predicted Mortality Probabilities for CABG	8.6 ± 9.2	8.0 ± 9.1	9.3 ± 9.3	0.4
Priority Indication				
Elective (%)	12.8	9.4	16.4	0.41
Urgent (%)	64.0	68.8	59.0	
Emergent (%)	23.2	21.9	24.6	

**Table 2:** Procedural Data for all Patients and Comparison of Bifurcation and Non-Bifurcation Cohorts

Variables	All	Bifurcation	Non-bifurcation	P-value
Number (N = 125)	125 (100%)	64 (51.2%)	61 (48.8%)	
IVUS use (%)	72.8	70.3	75.4	0.52
IABP use (%)	45.6	50.0	41.0	0.31
# of diseased vessels	2.4 ± 0.9	2.6 ± 0.7	2.2 ± 1.0	0.01
3 vessel disease (%)	63.2	73.4	52.5	0.02
Total stent count	3.0 ± 2.0	3.6 ± 2.0	2.3 ± 1.8	< 0.001
Left main stent count	1.7 ± 0.8	2.3 ± 0.7	1.1 ± 0.3	< 0.001
Heparin use (%)	78.4	84.4	72.1	0.10
IIb/IIIa inhibitor use (%)	26.4	35.9	16.4	0.01
Bivalirudin use (%)	41.6	32.8	50.8	0.04
Bifurcation stenting technique				
Angioplasty rescue (%)		30.2		
Crush stent (%)		15.9		
Culotte stent (%)		20.6		
Double barrel stent (%)		6.3		
Single stent (%)		3.2		
T stent (%)		20.6		
V stent (%)		1.6		
Y stent (%)		1.6		

### Bifurcation vs. non-bifurcation lesions

**Figure 2:**
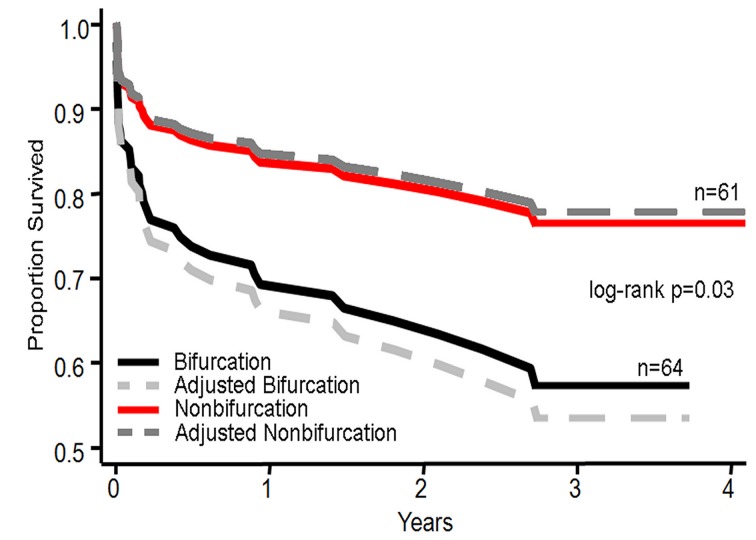
Risk adjusted Kaplan-Meier graph of survival for left main percutaneous coronary intervention by bifurcation or nonbifurcation. Risk adjusted Kaplan-Meier survival curve by for bifurcation and nonbifurcation with log-rank analysis adjusted for age, CHF, EF ≤ 35, cardiogenic shock, and creatinine > 2.

The 51.2% of cases involved bifurcation of the LMCA. Baseline data for bifurcation and non-bifurcation groups are presented in Table 1. The only significantly different characteristic was EF, with the bifurcation group having a higher mean EF than non-bifurcation group (52.2 vs. 46.1). Patients with lesions treated at the LMCA bifurcation had an increased mortality rate compared to non-bifurcation lesions (37.5% compared to 19.7%). Adjusting for covariates further strengthened the mortality difference between the two groups (Fig. 2). There was also a nonsignificant trend towards higher in-hospital mortality (12.5% versus 4.9%). There was no significant difference between cumulative MI or TVR rates. However, there was a non-significant trend of more revascularizations in the bifurcation group (26.6% compared to 16.4%).

### Priority designation

**Figure 3:**
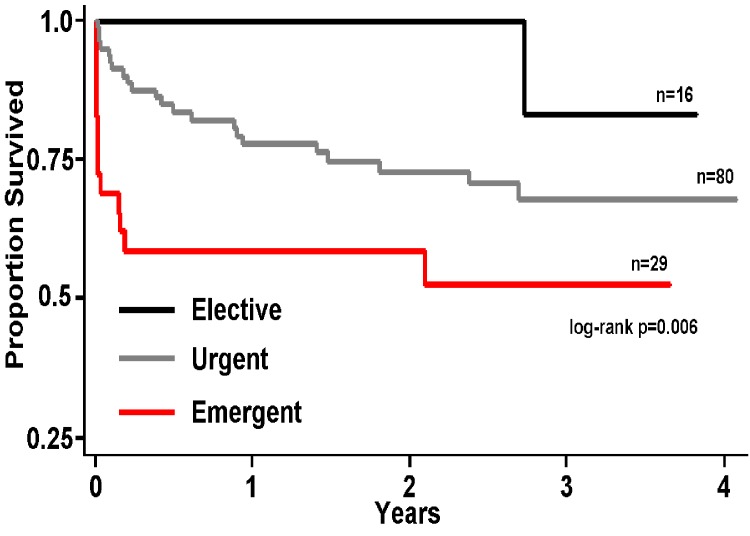
Kaplan-Meier graph of survival for left main percutaneous coronary intervention by priority. Kaplan-Meier survival curve by priority designation: elective, urgent, and emergent with log-rank analysis.

With increasing urgency, there was an overall decline in EF (elective 59.4%, urgent 51.6%, emergent 38.0%). Many emergent patients were in shock (62.1%) and not surgical candidates (89.7%). The urgent and emergent groups were more likely to have a positive troponin, acute coronary syndrome, ST-elevation MI and Non-ST-elevation MI, and be in cardiogenic shock. The more emergent procedures had higher risk with higher Parsonnet and Euroscores, and these procedures were more likely to have IABP utilized. Heparin and IIb/IIIa inhibitors were more likely to be utilized in the emergent cases, whereas bivalirudin was more likely to be used in elective cases. With increasing urgency of the procedure, there was an increase in the cumulative mortality (elective 6.3%, urgent 26.3%, emergent 44.8%) (Fig. 3). The emergent group had the highest 30-day mortality at 31.0%. Elective and urgent patients had in-hospital mortality comparable to expected mortality when looking at PCI risk models with the elective group at expected and the urgent group mortality 7% better than expected. The emergent group had 40% higher than expected mortality. There was no significant difference between myocardial infarction rates, TVR, or any revascularization between the three priority groups.

**Table 3:** Outcomes for all Patients and Comparison of Bifurcation and Non-Bifurcation Cohorts

Variables	All	Bifurcation	Non-bifurcation	P-value
Number (N = 125)	125 (100%)	64 (51.2%)	61 (48.8%)	
Mean follow-up (years)	1.8 ± 1.2	1.6 ± 1.2	1.9 ± 1.3	0.07
Mortality (%)	28.8	37.5	19.7	0.03
In-hospital mortality	8.8	12.5	4.9	0.14
Any revascularization (%)	21.6	26.6	16.4	0.17
TVR (%)	13.6	14.1	13.1	0.88
MI (%)	7.2	7.8	6.6	0.79
Mean time to death (years)	0.6 ± 0.8	0.6 ± 0.9	0.5 ± 0.8	0.62
Mean time to TVR (years)	0.6 ± 0.4	0.4 ± 0.2	0.8 ± 0.5	0.03
Mean time to any revascularization (years)	0.5 ± 0.4	0.4 ± 0.3	0.6 ± 0.6	0.19

### Analysis of risk factors for mortality

Cox proportional hazard regression models were utilized to analyze risk factors for mortality. Independent risk factors for mortality were found to be EF ≤ 35% (HR 2.44) and left main bifurcation lesions (HR 2.65) (Table 4). Other factors such as IABP use, high Parsonnet score and Euroscore, cardiogenic shock, positive troponin, and emergent priority had significant crude hazard ratios, but these did not result in independently significant ratios in the adjusted hazard ratio model. Of note, stent count, mitral regurgitation ≥ 2+, and acute coronary syndrome were not found to be significant risk factors.

**Table 4:** Crude and Adjusted Hazard Ratios

Risk Factor	Crude HR	95%CI	Adjusted HR	95% CI
EF < 35	2.33	(1.18 - 4.60)	2.44	(1.10 - 5.42)
LM bifurcation	2.07	(1.03 - 4.14)	2.65	(1.24 - 5.67)
IABP	3.73	(1.80 - 7.74)	1.67	(0.67 - 4.13)
Parsonnet Score	1.03	(1.02 - 1.04)	1.03	(1.00 - 1.05)
Euroscore	1.04	(1.02 - 1.06)	1.00	(0.97 - 1.03)
Cardiogenic Shock	3.00	(1.47 - 6.12)	0.64	(0.18 - 2.28)
Positive Troponin	3.27	(1.27 - 8.41)	1.90	(0.69 - 5.26)
Emergent	2.54	(1.29 - 5.02)	1.11	(0.39 - 3.17)

## Discussion

The purpose of the present study was to analyze patients who underwent PCI-stenting of the LMCA in a real world application. Our longest patient follow-up was 4 years with a mean follow-up of 1.8 years. Mortality was 10.4% at 30 days, 23.2% at 1 year, and 28.8% at 4 years. Cumulative TVR rate was 13.6%. Our study illustrates the viability of PCI-stenting as a treatment option in patients with LMCA stenosis.

Our survival rates are lower than that presented from long-term outcomes from CABG presented in the CASS (Coronary Artery Surgery Study) trial, where they had 10% mortality at 5 years and 26% at 10 years [6].

Several recent trials have presented outcomes on ULMCA PCI [24-27]. A recent meta-analysis of these randomized trials showed no significant difference in MACCE, death, MI when PCI compares with CABG at 1 year [28]. PCI results in higher repeat revascularization rates but lower stroke rates than CABG.

In comparison to these trials, our mortality rate is higher. Our population represents a real-world application of ULMCA PCI. Our population was at higher risk as evidenced by higher Parsonnet and Euroscores. Patients in our elective priority group had surgical risk score similar to those seen in the SYNTAX trial. This group also had similar cumulative mortality to that seen in SYNTAX at a rate of 6.3%. Our 4 year mortality of 28.8% compares favorably to recent large registry real world data mortality at 30 months of 42.7% [29].

In our study, bifurcation of the LMCA was found to be an independent predictor of mortality. A recent registry also found an increase in major adverse cardiac events at 2 years with patients with bifurcation lesions [30]. Bifurcation lesions treated with double-stent techniques had worse outcomes at 1 year. Single stent technique was utilized in 33.4% of patients in our study with bifurcation lesions. However, there was no significant difference in outcomes when compared to 2 stent techniques.

EF ≤ 35% was also found to be an independent risk factor for increased mortality. These patient characteristics help further risk stratify patients and may ultimately guide in patient selection for LMCA stenting.

### Limitations

This is an observational study. As such, we cannot determine cause-effect relationships and can only suggest associations. Another limitation is our modest sample size which is a direct result of PCI of the LMCA being a relatively rare intervention. This does decrease the power of analyses. Personal operator preference was used with each intervention resulting in variability in the techniques used to treat each lesion, number of stents, stent type, as well as use of IVUS. This makes the comparison of each case difficult and can lead to confounding. The degree and timing of complete revascularization was also not looked at. We also did not look at the types of stents used; while the great majority of stents were DES, some of the interventions utilized bare-metal stents. DES may result in improved outcomes compared to bare-metal stents [29, 31].

### Conclusions

PCI of the LMCA remains a viable treatment option for patients in the drug-eluting stent era. It extends options in patients whom are poor candidates for coronary artery bypass grafts. Furthermore, elective LMCA PCI in low-risk CABG patients results in good survival rates. LMCA PCI is associated with a high restenosis rate of 13.6%. EF ≤ 35% and LM bifurcation were independent risk factors for mortality. Further studies regarding appropriate patient selection as well as procedural techniques regarding revascularization should be done to better define which patients may derive the best benefit from ULMCA PCI.
